# A sampling optimization analysis of soil‐bugs diversity (Crustacea, Isopoda, Oniscidea)

**DOI:** 10.1002/ece3.1765

**Published:** 2015-12-17

**Authors:** Giuseppina Messina, Roberto Cazzolla Gatti, Angeliki Droutsa, Martina Barchitta, Elisa Pezzino, Antonella Agodi, Bianca Maria Lombardo

**Affiliations:** ^1^ Department of Biological, Geological and Environmental ScienceSection “M. La Greca”University of CataniaCataniaItaly; ^2^ Biological Diversity and Ecology LaboratoryBioClimLand Centre of ExcellenceTomsk State University (TSU)Lenin ProspektTomsk634050Russia; ^3^ Laboratory of Agricultural Zoology and EntomologyAgricultural University of AthensAthensGreece; ^4^ Department “G.F. Ingrassia”University of CataniaCataniaItaly

**Keywords:** Accumulation–rarefaction curves, Oniscidea, sampling optimization, species abundances, *α*‐, *β*‐diversity

## Abstract

Biological diversity analysis is among the most informative approaches to describe communities and regional species compositions. Soil ecosystems include large numbers of invertebrates, among which soil bugs (Crustacea, Isopoda, Oniscidea) play significant ecological roles. The aim of this study was to provide advices to optimize the sampling effort, to efficiently monitor the diversity of this taxon, to analyze its seasonal patterns of species composition, and ultimately to understand better the coexistence of so many species over a relatively small area. Terrestrial isopods were collected at the Natural Reserve “Saline di Trapani e Paceco” (Italy), using pitfall traps monthly monitored over 2 years. We analyzed parameters of *α*‐ and *β*‐diversity and calculated a number of indexes and measures to disentangle diversity patterns. We also used various approaches to analyze changes in biodiversity over time, such as distributions of species abundances and accumulation and rarefaction curves. As concerns species richness and total abundance of individuals, spring resulted the best season to monitor Isopoda, to reduce sampling efforts, and to save resources without losing information, while in both years abundances were maximum between summer and autumn. This suggests that evaluations of *β*‐diversity are maximized if samples are first collected during the spring and then between summer and autumn. Sampling during these coupled seasons allows to collect a number of species close to the *γ*‐diversity (24 species) of the area. Finally, our results show that seasonal shifts in community composition (i.e., dynamic fluctuations in species abundances during the four seasons) may minimize competitive interactions, contribute to stabilize total abundances, and allow the coexistence of phylogenetically close species within the ecosystem.

## Introduction

Biological diversity analysis is frequently used to describe both communities and regional species compositions (Magurran 1988, 2013) and is at the basis of many ecological models (MacArthur and Wilson 1967; Connell 1978; Stevens 1989). Evaluating biodiversity is not only important for drawing comparisons among sites (Cornell 1999), but also to estimate population sizes and to understand community compositions (Sanders and Entling 2010). Moreover, knowing which species occur in a given region is sometimes fundamental for conservation purposes and requires an accurate understanding of their distributions (Cardoso 2009). Maximizing species richness within a reserve is often a goal of conservation efforts (May 1988). Arthropods are a megadiverse group, and their abundance and diversity make it almost impossible to fully assess their richness, their functions in the ecosystem, and their geographical patterns (Ramos et al. 2001). The Oniscidea, or terrestrial isopods, are abundant and widespread components of the soil's fauna and play significant roles in soil ecology. They contribute in the regulation of organic matter and nutrients (Hassall and Sutton 1978; Sutton 1980; Zimmer et al. 2003) and are important elements of soil food webs, being themselves food sources for other arthropods (Vetter and Isbister 2006) as well as for vertebrates (Ben Hassine and Nouira 2009; Covaciu‐Marcov et al. 2012). Moreover, due to their biological and ecological characteristics, terrestrial isopods are used as biological indicators of heavy metal pollution (Paoletti and Hassall 1999) as well as of grassland habitats quality (Souty‐Grosset et al. 2005). Methods of data collection, including the sampling period, are highly important for studies focusing on terrestrial isopods diversity. Usually, collection of samples takes place over long periods of time, in order to assure an appropriate sample size for further analyses. As a consequence, the sampling procedure is generally time consuming and expensive in terms of materials and personnel (Sutherland 2006), while collecting very large numbers of specimens may reveal detrimental for conservation purposes. Even if a wide range of techniques is available for sampling isopods, these techniques need to be standardized to varying degrees to enable catches to be comparable over time or between areas (Sutherland 2006). This study tries to assess the optimum sampling effort and the seasonal timing necessary to maximize detected species diversity during data collection, while investing in fewer resources in terms of time, money, personnel, etc. Moreover, our research analyzes the spatiotemporal shifts in community composition in order to understand mechanisms allowing the coexistence of such a high number of Isopoda species in a relatively small area.

## Materials and Methods

### Sampling area

This study was carried out in the Natural Reserve “Saline di Trapani e Paceco” (Fig. S1), which includes a Special Protection Area (“SPA”, for the purposes of the European “Birds Directive” 79/409/CEE), an “Important Bird Area”, a RAMSAR‐Convention protected wetland, as well as a EU Site of Community Importance (“SCI”, ITA 01007). The reserve is located in the south of the Province of Trapani, on the west coast of Sicily (Italy); it covers a surface of 960 ha and consists of a flatland with sandy coasts and large wetlands.

### Sampling method

Data were obtained following a standardized sampling method. Pitfall traps were randomly distributed all over the area and consisted of 29 sampling units, grouped in five spatially disjoined replicates (Koivula et al. 1999; Lassau et al. 2005). Traps were monitored monthly all through a 24‐month period, from February 2008 to February 2010. The traps (diameter 10 cm, height 14 cm) were sunk into the ground, with their rims level with the soil surface, and were half‐filled with a saturated water/sodium chloride solution, in order to avoid the attractive effects of formalin and vinegar. Pitfall trapping is a widely used method (Pekár 2002), as it is time efficient, easy to use, and inexpensive, while it produces large species‐rich samples suitable for statistical analyses (Spence and Niemelä 1994). It is broadly recognized as a valid sampling technique for the soil invertebrate fauna in general (New 1999; Brandmayr et al. 2005) as well as for isopods in particular (Becker 1975; Fleugge and Levens 1977; Al‐Dabbagh and Block 1981; Caruso and Zetto Brandmayr 1983).

### Statistics

Data of species richness and of individual species abundances obtained by each sampling unit were pooled separately for the 2 years and the four seasons of each year (see Table 1). We analyzed data of *α*‐ and *β*‐ diversity by the software EstimateS (Colwell 2013). More in detail, for each species, we recorded the number of sampled individuals and evaluated the species richness of the community by Chao 1 index. For each assemblage, we calculated the relative dominance and McNaughton's dominance (which accounts for three most dominant species), as well as Shannon's and Simpson's inverse diversity and evenness. Classical Jaccard and Sorensen similarity measures were computed to better understand the complementarity of each coupled seasonal assemblage independently of each species' abundance (i.e., only on incidence data) and to disentangle the effects of dominance on the communities. We also calculated Whittaker's *β*W measure, which is widely recognized as the less affected by error among indexes of *α*‐diversity (Cazzolla Gatti 2014). We computed abundance‐based *β*‐diversity measures (such as Bray–Curtis, Chao‐Jaccard, and Chao‐Sorensen), to understand the influence on similarity of the distribution of individuals among species. Finally, we calculated the Morisita‐Horn index, which is known as the best performing abundance‐based index of complementarity (Magurran 2013). For distributions of species abundances and accumulation and rarefaction curves, we followed the approach proposed by Magurran (2004) or by Cazzolla Gatti (2014) to analyze changes in biodiversity over time.

**Table 1:** Species sampled in each season during the 2 years of sampling

Species	Spring 2008	Summer 2008	Autumn 2008	Winter 2008	Spring 2009	Summer 2009	Autumn 2009	Winter 2009
*Tylos ponticus* (Grebnicki, 1874)	●			●	●			
*Ligia italica* (Fabricius, 1798)			●					
*Armadilloniscus candidus* (Budde‐Lund, 1885)					●			
*Armadilloniscus ellipticus* (Harger, 1878)	●				●			
*Halophiloscia couchii* (Kinahan, 1858)	●	●	●	●	●	●	●	●
*Halophiloscia hirsuta* (Verhoeff, 1928)	●			●			●	
*Stenophiloscia glarearum* (Verhoeff, 1908)	●			●	●			
*Chaetophiloscia elongata* (Dollfus, 1884)	●	●	●	●	●	●	●	●
*Porcellionides pruinosus* (Brandt, 1833)			●		●		●	
*Porcellionides sexfasciatus* (Budde‐Lund, 1885)						●		
*Acaeroplastes melanurus* (Budde‐Lund, 1885)	●	●	●	●	●	●	●	
*Agabiformius lentus* (Budde‐Lund, 1885)	●	●	●	●	●	●	●	●
*Agabiformius obtusus* (Budde‐Lund, 1909)					●			
*Leptotrichus panzerii* (Audouin, 1826)	●	●	●	●	●	●	●	●
*Lucasius pallidus* (Budde‐Lund, 1885)	●				●			
*Mica tardus* (Budde‐Lund, 1885)	●	●			●			
*Porcellio albicornis* (Dollfus,1896)		●	●	●	●	●	●	●
*Porcellio laevis* (Latreille, 1804)	●	●	●	●	●	●	●	●
*Porcellio siculoccidentalis* (Viglianisi, Lombardo, Caruso, 1992)	●		●	●	●	●		●
*Armadillidium album* (Dollfus, 1887)				●	●		●	●
*Armadillidium badium* (Budde‐Lund, 1885)	●	●	●	●	●	●	●	●
*Armadillidium decorum* (Brandt, 1833)	●	●	●	●	●	●	●	●
*Armadillidium granulatum* (Brandt, 1833)	●	●	●	●	●	●	●	●
*Armadillo officinalis* (Dumeril, 1816)	●	●	●	●	●	●	●	●

We checked the statistical significance of differences in the distribution of abundances between the rank–abundance plots of the 2 years by Kolmogorov–Smirnov two‐sample test.

### Accumulation and rarefaction indexes

SACs (Species–area curves) are widely used in biodiversity research and can provide useful information to optimize sampling efforts and reduce resource wasting in future research. Although species accumulation curves, such as the abovementioned SACs, can be used to draw inferences about the diversity of a more fully censused assemblage (Gotelli and Colwell 2001), rarefaction curves allow to estimate richness at the abundance level of the smallest sample. For these reasons, we used rarefaction curves to better compare seasonal assemblages and speculate about their “real” richness, irrespectively of the sampled area. We used Coleman's type rarefaction curve, which estimates the number of species in samples, on the assumption that all individuals in all samples were randomly mixed (Chazdon et al. 1998). We used SACs also to compare the four seasons of each year.

### ECDF (Empirical Cumulative Distribution Function)

As, when comparing samples with high differences in richness, most plot types tend to overemphasize differences in richness, and curves of the richest sites become stretched in the low right corner of the graph (i.e., plotting data on k‐dominance graph), which makes them apparently more even, we represented abundance data with ECDF plots, which can better discriminate between assemblages by rescaling their ranks according to richness (Magurran 2013; Cazzolla Gatti 2014).

### Species viability (population dynamics)

Species viability (i.e., population dynamics according to abundances) of the eight seasons of both years was used to describe patterns of species compositions during time.

### SAD (Species abundances distributions)

Species abundances distributions are generally used to shed light on processes that determine the biological diversity of a species assemblage. Frequency distribution plots, where the number of species is displayed in relation to the number of individuals per species, permit to understand and compare dominance/evenness patterns. In these graphs, the mode usually falls on the lowest abundance class.

### Rank–abundance plot

Rank–abundance plots (also known as Whittaker's plots) are among of the most informative methods to evidence contrasting patterns of species richness and to highlight differences in evenness among assemblages.

## Results

A total of 25,690 individuals belonging to 24 species were collected. Table 1 shows a list of all the species sampled in each season.

**Figure 1:**
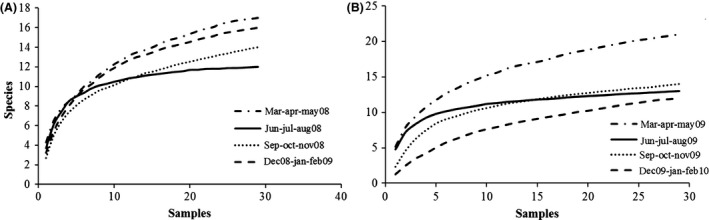
Species–area curves for the four‐season assemblages of the first (A) and second year (B). On the *x*‐axis is shown the cumulated number of samples (resampled 100‐fold), while the number of species is represented on *y*‐axis.

**Figure 2:**
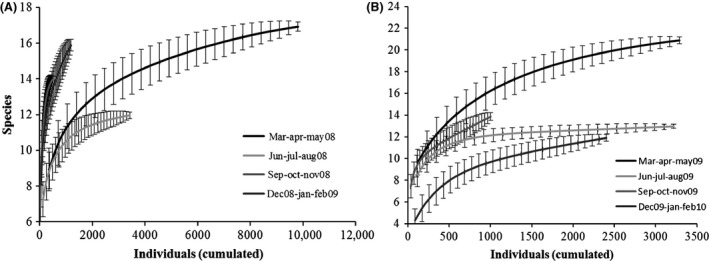
Coleman's rarefaction curves of the four seasons of the first (A) and second year (B). On the *x*‐axis is represented the cumulated number of individuals (resampled 100‐fold) and on the *y*‐axis the number of rarefied species.

**Figure 3:**
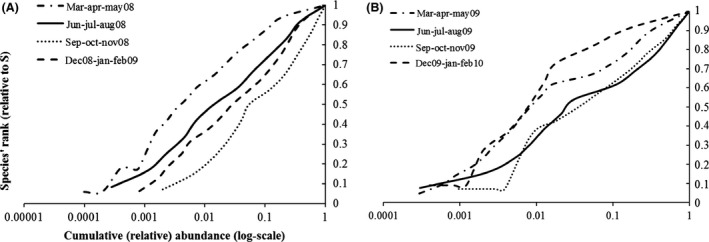
Empirical Cumulative Distribution Function plot of the four seasons of the first (A) and second year (B). On the abscissa are represented the cumulative relative abundances (rescaled on log10), while the species' rank (rescaled over richness) is shown on the ordinates.

**Figure 4:**
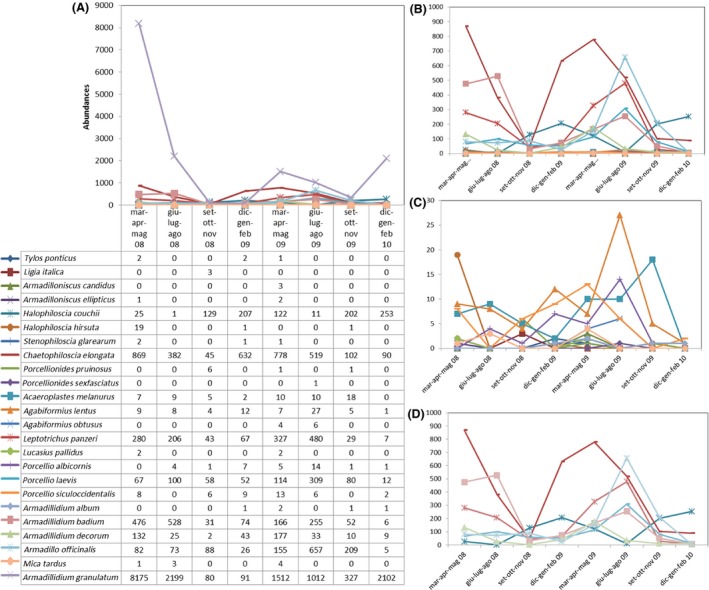
Species viability of (A) all species; (B) all species removing *Armadillidium granulatum* (the most abundant species); (C) rare (*n*_TOT_ ≤ 100); and (D) dominant species (*n*_TOT_ > 100) removing *A. granulatum* during the 2 years (eight seasons). On the *x*‐axis is represented the seasons and on the *y*‐axis the abundances.

The *α*‐ and *β*‐diversity indexes are, respectively, summarized in Tables 2 and 3, where the basic indicators (such as the number of species and the number of individuals) are shown together with dominance (relative dominance and McNaughton dominance), diversity and evenness indexes (Shannon's and Simpson's inverse diversity and evenness), and Chao 1. The SACs for each season of the 2 years are shown in Figure 1 and were calculated by plotting sampling units' increments against the number of species sampled, with 100 randomizations of sample units to obtain a smoothed curve. Accumulation and rarefaction curves are shown in Figure 2. The ECDF independence to richness is shown in Figure 3 for both years. For a clearer and more detailed analysis of population dynamics according to abundances, we separately plotted all the species (Fig. 4A), all but the most abundant one (i.e., *Armadillidium granulatum*, Fig. 4B), only the rare species (those with an annual total species abundance of *n* ≤ 100, Fig. 4C), and only the dominant species (having annual total abundance of *n* > 100) but excluding *A. granulatum* (Fig. 4D).

**Table 2:** *α*‐Diversity indexes

Season	S	N	Chao 1	Chao 1 SD	Shannon (H)	Simpson (D)	E_H_	E_D_	Relative dominance	McNaughton dominance
Spring _08	17	10157	17.67	1.31	0.81	1.52	0.286	0.089	0.80	31.24
Summer_08	12	3538	12	0.55	1.25	2.35	0.503	0.196	0.62	29.29
Autumn_08	14	501	14.5	1.32	2.05	6.42	0.777	0.458	0.26	19.76
Winter_08	16	1227	18.25	3.39	1.65	3.23	0.595	0.202	0.52	25.26
Spring _09	21	3406	22.5	2.29	1.70	3.73	0.558	0.178	0.44	25.61
Summer_09	13	3334	13	0.48	1.83	5.25	0.713	0.404	0.30	21.88
Autumn_09	14	1038	19.99	7.19	1.85	5.08	0.701	0.363	0.32	23.70
Winter_09	12	2489	13.5	2.60	0.60	1.38	0.241	0.115	0.84	32.74

**Table 3:** *β*‐Diversity indexes

Season 1	Season 2	Jaccard Classic	Sorensen Classic	Wittaker Bw	Chao‐Jaccard‐Raw Abundance‐based	Chao‐Sorensen‐Raw Abundance‐based	Morisita‐Horn	Bray–Curtis	S tot
Spring 2008	Summer 2008	0.611	0.759	1.241	0.996	0.998	0.957	0.503	18
Spring 2008	Autumn 2008	0.550	0.710	1.290	0.997	0.989	0.355	0.071	20
Spring 2008	Winter 2008	0.737	0.848	1.151	0.993	0.997	0.226	0.181	19
Summer 2008	Autumn 2008	0.733	0.846	1.154	0.969	0.984	0.448	0.170	15
Summer 2008	Winter 2008	0.647	0.786	1.214	0.988	0.994	0.315	0.307	17
Autumn 2008	Winter 2008	0.667	0.800	1.200	0.978	0.989	0.511	0.487	18
Spring 2009	Summer 2009	0.545	0.706	1.294	0.994	0.997	0.875	0.702	22
Spring 2009	Autumn 2009	0.591	0.743	1.257	0.990	0.995	0.803	0.403	22
Spring 2009	Winter 2009	0.571	0.727	1.273	0.992	0.996	0.781	0.600	21
Summer 2009	Autumn 2009	0.688	0.815	1.185	0.995	0.998	0.859	0.382	16
Summer 2009	Winter 2009	0.786	0.880	1.120	0.996	0.998	0.576	0.397	14
Autumn 2009	Winter 2009	0.733	0.846	1.154	0.980	0.990	0.630	0.375	15

**Figure 5:**
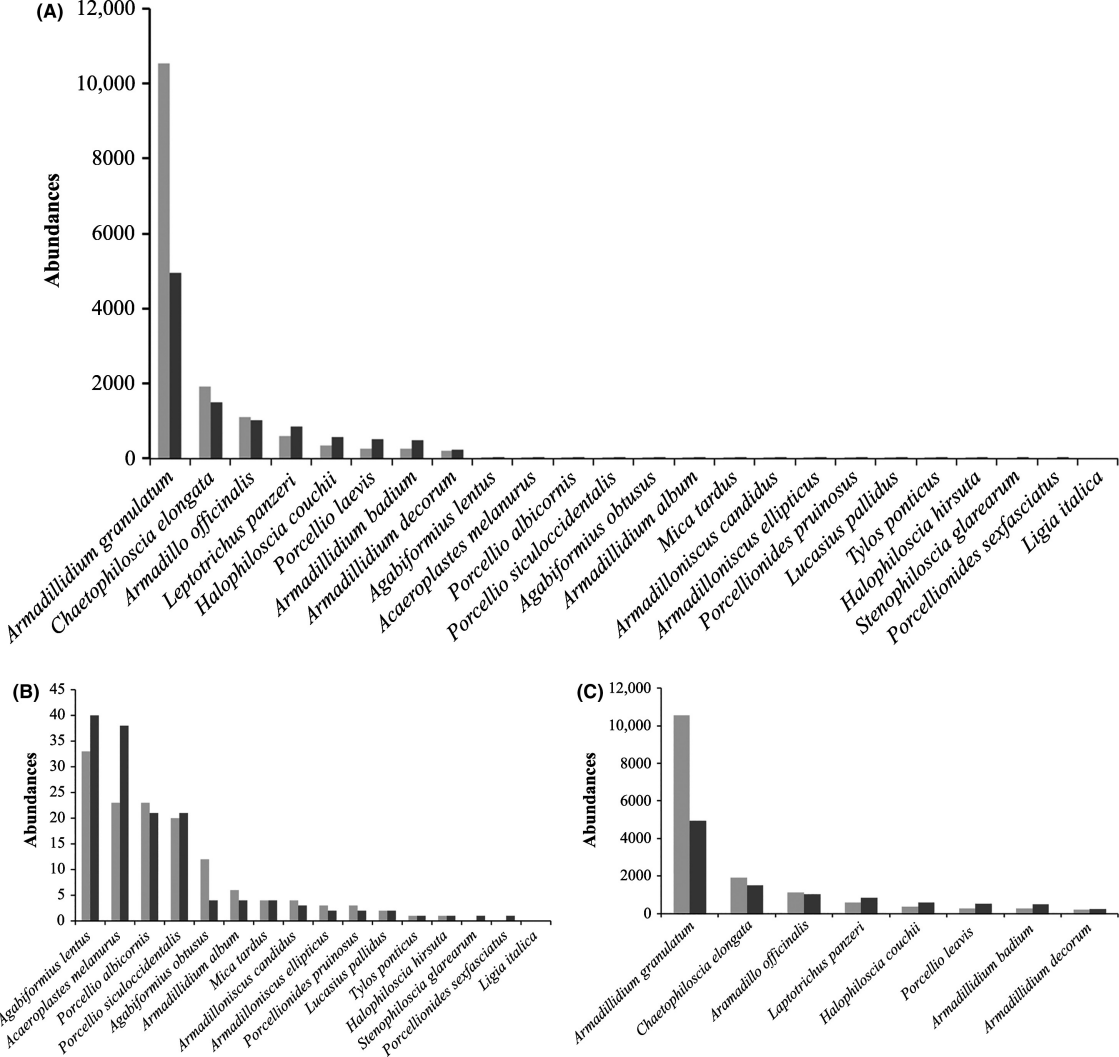
Species–abundance distributions of all species (A), rare species (*n* ≤ 200) (B), and dominant species (*n* > 200) (C) represented in the two‐year assemblages. Species are shown on the *x*‐axis and their order of abundance on the *y*‐axis.

Frequency distribution plots for all, rare (*n* ≤ 200 individuals/year collected) and dominant (*n* > 200) species are presented in Figure 5A–C, respectively. These plots show the highest frequency in the rare species class, as well as differences between the 2 years.

**Figure 6:**
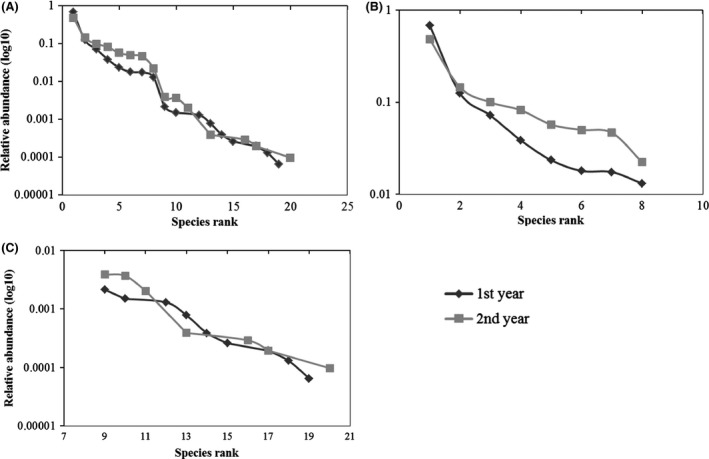
Rank–abundance plots of all species (A), dominant species (B) and rare species (C) observed in the 2 years of sampling. On abscissae is shown the species rank and the relative abundance of each species is shown on the ordinates (rescaled with the log 10).

As in the case of SADs, we plotted rank–abundance data for all, rare, and dominant species to better understand differences in species distributions occurring in each annual assemblage (Fig. 6A–C, respectively). On the base of these distributions, we also calculated Kolmogorov–Smirnov statistical tests.

## Discussion and Conclusion

Our research focuses on the study of the Oniscidea, a taxon already widely studied in the Mediterranean region, most particularly in Sicily (Caruso 1968, 1973a,b, 1974, 1976; Caruso and Lombardo 1976; Caruso et al. 1987; Messina et al. 2011), north Africa (Hamaїed‐Melki et al. 2010; Khemaissia et al. 2012a,b) and Greece (Alexiou and Sfentourakis 2013). The present work analyzes both the isopod diversity of the study area and the optimal approach capable to maximize our understanding of the contribution of species diversity while allowing to spend fewer resources for data sampling.

### α‐Diversity

As is shown in Table 2, in both sampling years, the highest species richness (S) occurs in spring (17 and 21 species, respectively). The highest total abundance (as value of N) is also shown to occur in the spring of both years (10,157 and 3406 specimens, respectively). In general, spring is the best growth season for most of the 24 species observed. According to the Chao 1 estimator (Table 2), in both years, the number of species expected to occur in the community is very close to the observed species richness (18.25 ± 3.39 expected vs. 17 observed, in 2008; 22.5 ± 2.29 vs. 21, in 2009), so that our sampling can be considered relatively complete. Since in 2008, the diversity of the general community is strongly influenced by the dominance of *Armadillidium granulatum* (8175 individuals found in spring, see Tables 1, 2 and Fig. 5A), the evenness for that season (spring 2008) is extremely low (E_H_' = 0.29 and E_D_ = 0.089), which is also reflected in the lowest values of both diversity indexes (H' and D(inv)). When in the autumn of 2008, the population of *A. granulatum* decreases (Figs. 5A, 5C), evenness increases and diversity peaks (Table 2). Cases of population explosions (Warburg 1993) of *Armadillidium vulgare* (Latreille, 1804) in North America (Hatch 1947), *A. granulatum* on Panarea (Caruso 1968), and *Armadillidium decorum* at Collesano (Italy) are well documented in the literature. In 2009, on the contrary, no particularly high dominance of one or a few species is observed, and the maximum diversity level is reached between summer (Simpson inv.) and autumn (Shannon). This latter result is due to the well‐known effects of highest abundances on Simpson index (which, in summer 2009, is influenced by a triple total number of individuals compared to that of the autumn 2009). Summarizing, in terms of richness, spring is the best season for the analyzed species, while, considering evenness values, diversity was maximum between summer and autumn (when dominance of the most abundant species decreases).

### β‐Diversity

Analyzing changes in *β*‐diversity values along time, by pairwise summing in turns two of the four seasons of each year (Table 3), in parallel with incidence indexes (presence/absence data only and ignoring abundances), for 2008 the best combination of the coupled seasons is spring–autumn (where both Jaccard and Sorensen indexes are lowest, which means less similarity and more diversity, Table 3). In 2009, in contrast, the best combination is slightly anticipated by one season (spring–summer), although *β*‐diversity remains high also in spring–autumn. Anyway, all these measures are strongly influenced by species richness and by sample sizes. To increase the reliability of our results, we also calculated Whittaker's (Bw) index (a *β*‐diversity incidence measure, Table 3), which provides values that are less influenced by errors and has fewer restrictions. This index confirms the best combinations of seasons shown by Jaccard and Sorensen indexes. Because our sampling cannot be considered totally complete (SACs were close to saturation but, apart from the case of summer, did not reach plateau Fig. 1) and differences in the number of individuals of the dominant species play a major role in abundance distributions (Fig. 5A–C), the abundance‐based measures of *β*‐diversity (Chao‐Sorensen, Chao‐Jaccard, and Bray–Curtis) gave contradictory results, so that they cannot be considered reliable indexes of diversity, in this case. The same considerations apply to the Morisita‐Horn index, which is strongly influenced by the abundance of the dominant species in the sample. In an attempt to provide the best possible representation of *γ*‐diversity, and eventually optimize time and resources, if we compare the highest number of species (S) collected in the coupled seasons with results shown by the indexes (Table 3), it appears that only the three incidence measures really reflect the highest values of S in each couple (20 in spring–autumn 2008 and 22 in spring–summer 2009).In summary, *β*‐diversity is maximized if samples are collected first during the spring and then between summer and autumn. The winter season provides the worst results when paired with any other season and should not be considered in this framework, at least whenever resources are limited and logistics are complex. Moreover, excluding the winter season does not significantly affect the sampling completeness of the community, as during the coupled seasons spring–summer/autumn, the number of collected species is close to the *γ*‐diversity values observed in both years (24 of *γ* vs. 20 of *β*, in 2008; 24 of *γ* vs. 22 of *β*, in 2009).

### Accumulation and rarefaction

The analysis of species areas curves (Fig. 1) shows that our sampling (five replicates of 29 sampling units) is very close to saturation in every season, and reaches complete plateau in the summer of both years. Also in both years, spring is the season that shows the highest values of species richness (as shown also by *α*‐ and *β*‐diversity analyses) irrespectively of the number of collected samples, whereas the winter of 2009/2010 shows the lowest increments. Indeed, three of the four species not collected during the latter season (*Tylos ponticus, Halophiloscia hirsuta, and Stenophiloscia glarearum*) live near the shoreline and their occurrence depends on the weather and the conditions of the sea (Vandel 1962), which were particularly harsh in the winter 2009/2010. The analysis of Coleman's rarefaction curves (Fig. 2) shows that if, to optimize the sampling effort, we consider the minimum number of individuals as our reference value, we get controversial results. This may be due to the large fluctuations of abundances during the year and to the frequency of oscillations in different years (see Figs. 5, 6). For instance, the low number of individuals recorded in the autumn 2008 was due to a strong decrease in the populations of the dominant species, which was not observed in 2009. This is the reason why, if we rarefy the number of individuals to a minimum, autumn shows the highest richness in 2008 but not in 2009 (when dominant species populations are more even – see populations dynamics).In summary, while accumulation curves (SAC) show that the sampling dimension utilized is adequate for a reliable representation of *γ*‐diversity, rarefaction in this case is inadequate to suggest an optimized sampling effort, being strongly influenced by abundances and, in our case, by those of only one or a few dominant species. Anyway, rarefaction offers valuable information on the minimum overall number of individuals needed to sample the highest richness in each season. What emerges, in fact, is that while less than 2000 individuals are enough to represent the richness of the community during summer, autumn, and winter, this number has to be increased in the case of spring.

### ECDF

The ECDF affords a better understanding of the distribution of species abundances, it is less influenced by richness, and it is also more reliable than Whittaker plots. ECDF shows that in 2008 (Fig. 3A), spring accounts for the highest number of rare species (bottom left part of the curve), but at the same time also for the lowest (central part of the curve with a gradual slope, with less verticality) and more evenness, as shown by *α*‐diversity analysis. In 2009 (Fi g. 4B), instead, winter evenness is the lowest because of the absence of some species, while total abundance remains high, as also shown by *α*‐ and *β*‐diversity analyses, but the number of rare species is lower than in spring and summer.

### Species viability (population dynamics)

Analyzing species population dynamics (Fig. 4), a general trend of population decrease emerges in the autumn of both years and for all species, although, apart from *Porcellio laevis* (in the dominant group), the rare species seems to be less prone to a reduction, if compared with dominant species. These fluctuations can explain the observed patterns of *α*‐ and *β*‐diversity: The dominant species generally decline in autumn, whereas rare species are stable in this season, and even if their populations fluctuate, often disharmoniously so, during the rest of the year. Species that dominate the growing season of the first year seem to reduce their dominance in the next year, and vice versa. At the same time, the abundance of the dominant species in the first year (*A. granulatum*) is higher than that in the second year, whereas, apart from *A. granulatum* and as also shown by *α*‐ and *β*‐diversity, the abundances of both rare and dominant species are higher in the second year, but with a lower number of species. In agreement with Shimadzu et al. (2013), our results show that spatiotemporal shifts in community composition can minimize competitive interactions, increase some biodiversity values, and help stabilize total abundances.

### SAD

To gain better understanding of the patterns shown by *α*‐ and *β*‐diversity and by population dynamics, we analyzed SADs also as species abundance histograms and Whittaker plots, by splitting the data into three categories: total abundances, dominant abundances, and rare abundances. The simple plotting of species and abundances (Fig. 5) shows a low number of dominant species (8) compared to the high number of rare species (16) in both years (Fig. 5A). In 2008, the three most abundant species show even higher dominance than in 2009 (Fig. 5C). The other five dominant species may suffer the dominance of the three most abundant ones, as they show reduced populations in the first year, but not in the second, when the three most dominant species accounted for a lower number of individuals (Fig. 5C). Figure 5B shows that, in contrast, the abundances of rare species (*n* ≤ 200) are higher when populations of the three most dominant species are lower (second year). Moreover, the number of rare species increases when dominance is reduced (second year, Fig. 5B). Dividing species and their relative abundances into three groups (total, dominant, and rare), Whittaker plots show that while both communities maintain the same general distribution in both years (Fig. 6A), the dominant species (*n* > 200) plot (Fig. 6B) confirms a less even distribution and an higher dominance (steeper slope) in the first year (2008) as compared to the second one (2009). The rare species (*n* ≤ 200) plot (Fig. 6C), instead, shows no visual difference in distribution between years. Anyway, the cumulative two‐year distributions show no significant statistical difference (D21,23 = 0.201 *P* > 0.01) when checked with Kolmogorov–Smirnov test and this confirms that the distribution patterns of the two communities in 2008 and 2009 were similar and that our results can be considered valid for both years. This closer look at the distributions of dominant and rare species allowed us to get a detailed picture of the assemblages of the analyzed communities. It also confirms observation derived from *α*‐ and *β*‐diversity measures, which we made with the aim to provide advice on how to reduce the sampling effort, as well as to save time and economic resources, without losing information when analyzing soil‐bugs biological diversity.

## Conflict of Interest

None declared.

## Supporting information


**Figure S1**. Study site.
