# Supplementary figures and images for: A sampling optimization analysis of soil‐bugs diversity (Crustacea, Isopoda, Oniscidea)
Source: Ecol Evol. 2015 Dec 17;6(1):191–201. doi: 10.1002/ece3.1765 (PMC4716513; doi:10.1002/ece3.1765)

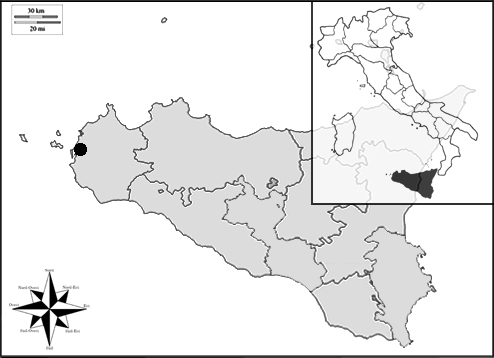

Supplement: Supplementary file 1 — Figure S1. Study site. [file ECE3-6-191-s001.jpg]
